# Estimating offsets for avian displacement effects of anthropogenic impacts

**DOI:** 10.1002/eap.1983

**Published:** 2019-08-30

**Authors:** Jill A. Shaffer, Charles R. Loesch, Deborah A. Buhl

**Affiliations:** ^1^ U.S. Geological Survey Northern Prairie Wildlife Research Center 8711 37th Street SE Jamestown North Dakota 58401 USA; ^2^ U.S. Fish and Wildlife Service Habitat and Population Evaluation Team 3425 Miriam Avenue Bismarck North Dakota 58501 USA

**Keywords:** anthropogenic disturbance, avoidance, biodiversity offsets, decision‐support tools, displacement, energy infrastructure, grassland birds, landscape‐level conservation goals, mitigation, oil development, waterfowl, wind energy

## Abstract

Biodiversity offsetting, or compensatory mitigation, is increasingly being used in temperate grassland ecosystems to compensate for unavoidable environmental damage from anthropogenic developments such as transportation infrastructure, urbanization, and energy development. Pursuit of energy independence in the United States will expand domestic energy production. Concurrent with this increased growth is increased disruption to wildlife habitats, including avian displacement from suitable breeding habitat. Recent studies at energy‐extraction and energy‐generation facilities have provided evidence for behavioral avoidance and thus reduced use of habitat by breeding waterfowl and grassland birds in the vicinity of energy infrastructure. To quantify and compensate for this loss in value of avian breeding habitat, it is necessary to determine a biologically based currency so that the sufficiency of offsets in terms of biological equivalent value can be obtained. We describe a method for quantifying the amount of habitat needed to provide equivalent biological value for avifauna displaced by energy and transportation infrastructure, based on the ability to define five metrics: impact distance, impact area, pre‐impact density, percent displacement, and offset density. We calculate percent displacement values for breeding waterfowl and grassland birds and demonstrate the applicability of our avian‐impact offset method using examples for wind and oil infrastructure. We also apply our method to an example in which the biological value of the offset habitat is similar to the impacted habitat, based on similarity in habitat type (e.g., native prairie), geographical location, land use, and landscape composition, as well as to an example in which the biological value of the offset habitat is dissimilar to the impacted habitat. We provide a worksheet that informs potential users how to apply our method to their specific developments and a framework for developing decision‐support tools aimed at achieving landscape‐level conservation goals.

## Introduction

Biodiversity offsetting is the term applied to compensation for unavoidable environmental damage from anthropogenic development, in which the goal is to achieve a net neutral or positive outcome through the restoration of degraded habitat or the reconstruction of new habitat, thus generating ecologically equivalent gains elsewhere (Gibbons and Lindenmayer 2007, Kiesecker et al. 2009, Doherty et al. 2010, Maron et al. 2012). “Averted loss” is the term applied to the protection of existing habitat from any future forms of development and is considered a compensated form of net loss (Curran et al. 2014). Biodiversity offsets, also known as compensatory mitigation, conservation banking, off‐site mitigation, or habitat set‐asides, are considered the last step in the mitigation hierarchy of avoid, minimize, restore, and offset (SARA 2010, Code of Federal Regulations 2002). Offsets are advantageous to industry in providing a way to calculate environmental risk, to governmental regulators in providing a way to encourage industry to act responsibly in the absence of legislation, and to conservation organizations in providing a way to align conservation goals with government and business planning (Kiesecker et al. 2009).

Biodiversity offsetting historically has been applied to wetland habitats (McKenney and Kiesecker 2010) and, more recently, to terrestrial habitats impacted by energy development (Doherty et al. 2010, Kiesecker et al. 2010). The impediments to implementation in the energy sphere, such as the development of reliable and biologically based currencies for estimating the sufficiency of offsets and a landscape‐scale framework for applying the offsets (Doherty et al. 2010), will need to be addressed as societal demand for increased energy production grows. A heightened call for energy independence as an issue of national security in the United States continues to increase demand for domestic energy production from conventional, unconventional (e.g., gas shale, oil sands) and renewable sources (EO No. 13783, 2017). Although the United States has been a net energy importer since 1953, the country is expected to become a net energy exporter by 2022 and in 2018 became the largest global producer of crude oil (USEIA 2018). In Canada, high oil prices have driven the accelerated extraction of bitumen (oil sands) and thus, the rapid expansion of the oil and gas sector within central Canada (Copeland et al. 2011). As worldwide demand for energy grows, the proliferation of energy infrastructure has inevitably begun to encroach upon remaining wildlife habitat (McDonald et al. 2009). Projected areas of energy production growth in both the United States and Canada overlap with what remains of native temperate grasslands in these countries. Although the temperate grassland ecosystem provides critical habitat for breeding birds, this ecosystem is among the most endangered on Earth (Hoekstra et al. 2005) and has been negatively affected by the habitat loss and fragmentation accompanying energy development (McDonald et al. 2009). Energy development is an additional stressor to other forms of grassland loss, including loss due to conversion to agriculture (Lark et al. 2015) and urbanization (Marzluff 2001). As the populations of many species of grassland birds continue to decline (Sauer et al. 2013), providing compensatory habitat, or offsets, to counteract biodiversity loss from recently developed habitat is imperative to stop or reverse decades‐long population declines. Although challenging, developing a standardized methodology to define and therefore calculate biologically meaningful equivalent values for the loss of functional habitat provides a practical starting point for the offsetting process.

Energy production decreases the value of temperate grasslands to migratory birds through changes that affect avian mortality rate, reproductive success, and behavior. Avian mortality occurs from collisions with infrastructure such as turbine blades, transmission lines, and vehicles, as well as mortality from burning in natural gas flares and drowning in oil pits (Arnett et al. 2007, Loss et al. 2015). Avian habitat is lost to construction of infrastructure, including to foundations that support oil and gas wells, compressor stations, turbines, extensive road systems, and worker housing (McDonald et al. 2009, Fargione et al. 2012, Thompson et al. 2015). Coal and solar facilities remove nearly all of the natural habitat within their area of impact (McDonald et al. 2009). Habitat loss is an especially pernicious impact of energy construction because it creates anthropogenic edge and landscape fragmentation, which are the primary causes of species endangerment in North America (Wilcove et al. 1998, MEA 2005). Many species of grassland birds respond negatively to a reduction in area of grasslands caused by landscape fragmentation, with the accompanying increase in edge habitat and distance between the next available grassland patch (Ribic et al. 2009). Impacts to grassland birds from reduced patch size and increased edge inherent to grassland fragmentation result in lower avian abundance and reproductive success, such as complete abandonment of nests or increased predation of eggs, nestlings, or adults, as well as increased brood parasitism (Bakker et al. 2002, Herkert et al. 2003, Davis et al. 2006, Sliwinski and Koper 2012). Independent of habitat loss, energy development may also cause lowered avian reproductive success (Mahoney and Chalfoun 2016, Yoo and Koper 2017) and alteration of hydrology that can impact waterfowl abundance (Lange et al. 2018).

Energy development is also responsible for the behavioral avoidance, or displacement, of birds from suitable habitat near energy infrastructure; displacement has been found in several taxonomic groups that rely on grasslands for breeding habitat, including waterfowl (Winkelman 1992, Loesch et al. 2013, Lange et al. 2018), passerines (Johnson et al. 2000, Thompson et al. 2015, Shaffer and Buhl 2016), shorebirds (Pearce‐Higgins et al. 2012, Niemuth et al. 2013, Sansom et al. 2016), raptors (Pearce‐Higgins et al. 2009, Garvin et al. 2011), and upland game birds (Winder et al. 2014, Dupuie 2018). Behavioral displacement is typically measured as a decrease in avian density measured over distance from energy infrastructure (sensu Shaffer and Buhl 2016). Not all individuals of a species avoid energy infrastructure (Loesch et al. 2013, Shaffer and Buhl 2016), and few researchers have tackled the question of whether the reproductive success of individuals that remain is lowered due to stressors such as increased noise and human activity or change in predator composition (Hatchett et al. 2013, McNew et al. 2014, Mahoney and Chalfoun 2016, Yoo and Koper 2017). For individuals that do not settle near energy infrastructure, potential extended searches for suitable breeding habitat are energetically demanding and reduce the available energy that birds can allocate to reproductive activities (Pianka 1976). As breeding habitat becomes ever more scarce, birds are forced to settle into increasingly smaller patches of suboptimal grassland constrained by issues of area sensitivity and landscape fragmentation, and in which inter‐ and intraspecies competition and chance of predator depredation may be higher (Ribic et al. 2009). Reduced survival and lower reproductive success ultimately lead to population declines for species already in crisis (Brennan and Kuvlesky 2005), and thus, biodiversity offsetting takes on ever‐greater importance.

One challenge of biodiversity offsetting is locating offset sites with equivalent biological value to impact sites, which can be defined in numerous ways by individual practitioners and will be defined in this paper in terms of avian density. For birds, this issue is compounded by a number of factors that influence avian occupancy of any particular habitat patch. Land use determines occupancy; for example, some avian species prefer grasslands that have been grazed, others do not (Kantrud and Kologiski 1982). The location of the impact site relative to a species’ breeding range (i.e., at the periphery vs. at the core) may impact avian occurrence and density (Niemuth et al. 2012), as may moisture patterns (Niemuth et al. 2008) and elevation (Niemuth et al. 2017). The vegetation composition and configuration of adjacent habitat patches in the surrounding landscape matrix affect the avian occupancy of any particular habitat patch (Niemuth et al. 2017), as well as that patch's ability to maintain evolutionary processes that in turn, contribute to functional landscapes (i.e., ones that provide for population growth; Bruggeman et al. 2005). Furthermore, the acknowledged uncertainty factors inherent in biodiversity offsetting—time lags, failure to persist, measurability issues—are compounded at the landscape level by piecemeal, site‐level projects (Moreno‐Mateos et al. 2015). Because of these challenges, conservation scientists advocate a landscape‐level approach to identifying areas appropriate as offset habitat. Bruggeman et al. (2005) combined elements of ecological, evolutionary, and economic theory to inform a method for calculating biodiversity‐offset credits (i.e., the landscape equivalency analysis) that accounts for organismal abundance, landscape spatial structure such as patch size and connectivity distances, and genetic information such as genetic divergence. In the development‐by‐design concept espoused by Kiesecker et al. (2010), the authors describe a systematic approach whereby a map of priority areas for sensitive avian species serves as the foundation to assess impact to biodiversity of the particular locations of anthropogenic developments. Significant overlap between a development's infrastructure footprint and high‐conservation priority areas translate to higher offset costs, to the extent that a developer may decide to first avoid the area, and if not that, then to minimize the impact. For future developments in which a physical location has yet to be determined and the target resources are readily available outside of conservation priority areas, the development‐by‐design concept has merit in its ability to forecast impacts before pre‐project development input cost is high by steering developers away from areas with potentially high offset costs. Bruggeman et al. (2005) and Kiesecker et al. (2010) propose that the biological value of any particular habitat patch depends on its placement within the larger landscape matrix.

We used the results from research into behavioral avoidance of breeding waterfowl and grassland birds to wind facilities to calculate a percent displacement value, that is, the percentage of birds that avoid the area within a defined distance from wind infrastructure. We identified a process to quantify the amount of suitable breeding habitat required by these displaced birds. The objectives of our study were to (1) develop a method that estimates the amount of habitat needed to provide equivalent biological value for displaced avifauna, based on the ability to define five metrics: impact distance, impact area, pre‐impact density, percent displacement, and offset density, which we have termed the avian‐impact offset method, (2) provide percent displacement values for waterfowl and grassland birds, (3) provide examples of the method for cases where the offset habitat is of equivalent biological value as the impacted habitat, as well as where the offset habitat is not equivalent, using examples for wind and for oil infrastructure, (4) provide a worksheet that informs potential users how to apply our method to their specific developments, and (5) present a framework for developing decision‐support tools to inform landscape‐level conservation decisions for biodiversity offsetting. To demonstrate our method, we draw on examples from energy development because we have data for these examples, but provided that data are available to populate the metrics required for our method, it is applicable to other forms of anthropogenic development, such as agricultural conversion, urbanization, and construction of transportation infrastructure.

## Methods and Results

### Avian‐impact offset method metrics

Application of the avian‐impact offset method requires knowledge of four metrics (i.e., impact distance, impact area, pre‐impact density, percent displacement) for the impact site and one metric for the offset site (i.e., offset density). Each is defined as follows.

*Impact distance* is the linear distance for which infrastructure influences bird behavior, that is, the maximum distance from the infrastructure at which displacement has been shown. This distance can be estimated from scientific knowledge of bird behavior, as in our example for waterfowl based on Loesch et al. (2013) and our example for grassland birds based on Shaffer and Buhl (2016), or from conducting field‐based research aimed at obtaining this knowledge.

*Impact area* is a function of *impact distance* that indicates the spatial extent of habitat affected by development and within which some birds are assumed to be displaced. In the case of waterfowl, the number of wetlands or area of wetlands (measured using any land‐area metric, such as hectares or acres) circumscribed by the *impact distance* is the *impact area*. In the geography in which our example occurs, the Prairie Pothole Region (PPR) of the United States, 86% of the 3.4 million wetland basins mapped by the United States Fish and Wildlife Service (USFWS) National Wetlands Inventory (NWI) program are ≤0.80 ha, and thus NWI provides a measurable unit of number or area of wetlands. In the case of grassland birds, the entire expanse of grassland habitat circumscribed by the *impact distance* is the *impact area* and can be measured using any land‐area metric. The units for the *impact area* must match the units used for the *pre‐impact density*, as explained further in the description of *pre‐impact density*.

*Pre‐impact density* is a biological metric of density of breeding bird pairs within the impact site but before the impact occurs. It is calculated as the number of bird pairs relative to the amount of habitat in the impact site (e.g., pairs per wetland for waterfowl, pairs per hectare for land‐based birds). *Pre‐impact density* can be estimated using a variety of sources, including field‐based surveys or published sources of avian density within the relevant habitat and geographic location. It is important that the area units within the density match the units used for *impact area*. For example, for waterfowl, if the *impact area* is measured in number of wetlands, the density must then be measured as pairs per wetland. Additionally, because the number of breeding duck pairs is not a constant per unit wetland area, the size, location, and class of wetland also must be considered (Reynolds et al. 2006). For grassland birds, if the *impact area* is measured in hectares, then the density must be measured as pairs/ha. For situations in which published sources for *pre‐impact density* are not available, users of the avian‐impact offset method may need to conduct field‐based surveys for their target species, habitat, and geographic location.

*Percent displacement* is the percentage of bird pairs within the impact site that are reduced as the result of energy development or other sources of anthropogenic disturbance relative to the number of bird pairs that would be present in the absence of the disturbance. Estimates of *percent displacement* for several avian species are currently available for energy‐related disturbances (including but not solely roads) (Table 1, Table 2; Pearce‐Higgins et al. 2009, Garvin et al. 2011, Pearce‐Higgins et al. 2012, Thompson et al. 2015, Sansom et al. 2016). For situations in which published sources for *percent displacement* are not available, users of the avian‐impact offset method may need to conduct field‐based surveys for their target species, habitat, and geographic location, or use well‐defined assumptions based on existing information.

**Table 1:** Percent difference of Mallard (*Anas platyrhynchos*), Northern Pintail (*A. acuta*), Gadwall (*Mareca strepera*), Blue‐winged Teal (*Spatula discors*), and Northern Shoveler (*S. clypeata*) predicted breeding duck pair abundance between estimates for the median seasonal wetland size (0.2 ha) in the Kulm‐Edgeley (KE) and Tatanka (TAT) wind facilities in North Dakota and South Dakota, USA, relative to estimates for reference sites without wind development (see Loesch et al. 2013)

Site and year	Blue‐winged Teal		Mallard		Gadwall		Northern Pintail		Northern Shoveler	
	No. pairs	Change (%)	No. pairs	Change (%)	No. pairs	Change (%)	No. pairs	Change (%)	No. pairs	Change (%)
KE										
2008	214	0	218	−27	157	−20	58	−43	55	−13
2009	180	−6	146	−34	104	−32	51	−52	59	−9
2010	221	−36	157	−29	75	−56	71	−38	67	−28
TAT										
2008	893	−7	552	12	506	10	276	30	252	−5
2009	398	−22	197	−23	172	−9	116	−34	99	−33
2010	726	−20	271	−13	237	8	196	−21	202	−28

*Notes*

Negative values indicate lower pair estimates for wetlands in the wind development sites relative to reference sites. The average percent displacement for the five species equals −18%. This average was calculated by first computing an average percent change and total number of pairs for each species separately. Then, a weighted average of the average percent change for the five species was computed using the total number of pairs for each species as a weight.

**Table 2:** Percent displacement by distance category and year post‐treatment for eight grassland bird species across three wind facilities placed in grazed mixed‐grass prairies in North Dakota, USA (Acciona Tatanka Wind Farm and NextEra Energy Oliver Wind Energy Center) and South Dakota, USA (NextEra Energy SD Wind Energy Center), 2003–2012^a^

Years post‐treatment^b^	<100 m	100–200 m	200–300 m	Average
1	51.54 (15.51)	27.05 (15.22)	5.69 (14.05)	17.91 (9.47)
2	43.86 (14.44)	42.12 (14.51)	32.32 (13.48)	36.86 (9.06)
3	57.30 (9.77)	41.38 (9.32)	39.48 (8.74)	42.09 (5.87)
5	59.85 (10.03)	58.03 (10.04)	48.45 (9.23)	52.91 (6.22)

*Notes*

Values are means with SE in parentheses. Average column is a weighted average of the percent displacement values for the 3 distance bands in that row; areas of the distance bands were used as the weights.

a Average percent displacement values are based on the predicted densities per 100 ha from the ANOVA models in Shaffer and Buhl (2016). The eight grassland bird species are Upland Sandpiper (*Bartramia longicauda*), Savannah Sparrow (*Passerculus sandwichensis*), Vesper Sparrow (*Pooecetes gramineus*), Grasshopper Sparrow (*Ammodramus savannarum*), Clay‐colored Sparrow (*Spizella pallida*), Chestnut‐collared Longspur (*Calcarius ornatus*), Western Meadowlark (*Sturnella neglecta*), and Bobolink (*Dolichonyx oryzivorus*) (Shaffer and Buhl 2016).

b No data were gathered for 4‐yr post‐treatment.

*Offset density* is the biological metric of density of breeding bird pairs at potential offset sites and is necessary to assure comparable biological value at the offset site. *Offset density* can be estimated using a variety of sources, including field‐based surveys or published sources of avian density within the relevant habitat and geographic location.

### Avian‐impact offset method calculation

We first computed the number of bird pairs within the impact site from *impact area* and the estimate of *pre‐impact density*.

$$\begin{array}{cc} & \text{Bird Pairs Within Impact Site}\\ & =\text{Impact Area}\times \text{Pre‐Impact Density} \end{array}$$

Second, we used the *percent displacement* value to calculate the number of bird pairs predicted to be displaced:

$$\begin{array}{cc} \text{Bird Pairs Displaced}=\; & \text{Bird Pairs Within Impact Site}\\ & \;\;\times \text{Percent Displacement} \end{array}$$

Third, we calculated the amount of habitat necessary for displaced pairs relative to the density of pairs at potential offset sites:

Offset Area=Bird Pairs Displaced/Offset Density

The units for this area will be expressed in the area units used in the offset density measurement. For example, for land‐based birds for which the density in the offset site may be estimated as pairs/ha, this estimate of habitat needed will be hectares. However, for waterfowl, for which the density estimate may be pairs per wetland, the estimate of habitat needed will be number of wetlands. In this case, the area of wetlands needed also could be estimated using an average restored wetland size as:

$$\begin{array}{cc} \text{Wetland Area}=\; & \text{Offset Area}\\ & \;\;\times \text{Average Restored Wetland Size} \end{array}$$

In the situation in which equal biological value (i.e., equal avian densities) for both the impact and offset site is determined, density values cancel out in the equations, and the amount of habitat needed for displaced bird pairs can be estimated as

$$\begin{array}{c} \text{Offset Area}=\text{Impact Area}\times \text{Percent Displacement} \end{array}$$

This would most likely occur in situations where impact habitat and offset habitat are of the same habitat type (e.g., both native prairie in the same geographical setting and topography under the same land use).

**Figure 1:**
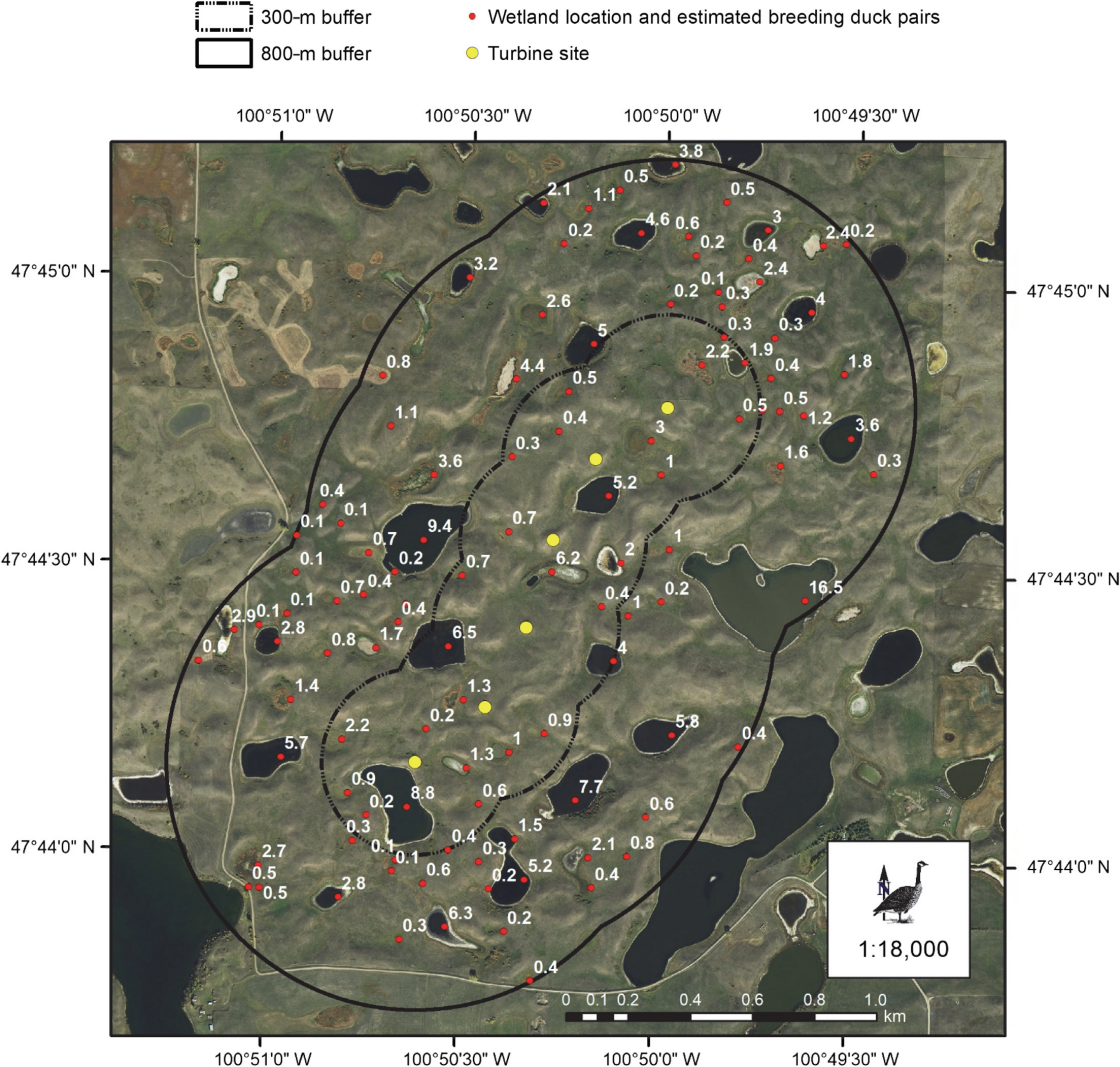
A hypothetical six‐unit string of wind turbines is placed in a grassland–wetland complex landscape typical of the native prairie landscape in North Dakota, USA. Turbines are, on average, 287 m apart. The 300‐m buffer zone represents the distance at which the density of breeding grassland bird pairs increasingly declined compared to reference sites. The area within that buffer zone is 112 ha. The 800‐m buffer zone represents the distance at which an average 18% displacement of waterfowl occurs. The area within that buffer zone is 431 ha. White numbers are the average number of waterfowl pairs per basin as predicted by United States Fish and Wildlife Service pair‐prediction models from the Four‐Square‐Mile Surveys (Reynolds et al. 2006).

In the examples below, we demonstrate how we used data from previously published research of breeding waterfowl displacement at wind facilities (Loesch et al. 2013) and grassland birds (Shaffer and Buhl 2016) to apply the avian‐impact offset method at a hypothetical wind facility located within the PPR in central North Dakota, USA (Fig. 1). For grassland birds, we will provide an example in which the offset habitat is deemed equal to the impact habitat in value, as well as an example in which it is not equal. We also provide one example in which we apply our method to a hypothetical oil‐extraction facility within the PPR. An accompanying worksheet provides a template to practitioners of our method in how to calculate the behavioral impacts of developments of interest to them (Appendix S1: Table S1). Furthermore, we supply decision‐support tools developed from spatial models that direct the delivery of biodiversity offsets to geographical locations where conservation benefits will be maximized after factoring in landscape‐level considerations.

### Example with waterfowl and wind infrastructure

We used a scenario in which a six‐unit turbine string with 287‐m turbine spacing is to be constructed in native prairie in North Dakota (Fig. 1). For waterfowl in the hypothetical wind facility, we used weighted average percent displacement for five duck species (Table 1), value of wetlands to breeding waterfowl (Reynolds et al. 2006), and characteristics of previously drained wetlands restored by the USFWS Partners for Fish and Wildlife Program in the North Dakota and South Dakota portion of the United States PPR (Loesch et al. 2012).

Consistent with the study design of Loesch et al. (2013), in which the objective of the study was to determine whether displacement existed and not to determine the zone of influence, we used an *impact distance* of 800 m. An 800‐m buffer was used because wetlands within that distance buffer would allow at least one turbine to be within the generalized home range of a breeding female Mallard (*Anas platyrhynchos*; Cowardin et al. 1988). For this hypothetical wind facility, the *impact area* encompassed 109 wetlands.

*Pre‐impact density* was determined using information from the Four‐Square‐Mile Breeding Waterfowl Survey (Cowardin et al. 1995), which has been used to develop pair‐prediction models for five species of dabbling ducks (i.e., Blue‐winged Teal [*Spatula discors*], Northern Shoveler [*S*. *clypeata*], Gadwall [*Mareca strepera*], Mallard, Northern Pintail [*A. acuta*]), and for four wetland classes (i.e., temporary, seasonal, semipermanent, and lake; Reynolds et al. 2006). We applied updated pair‐abundance models developed in 2012 to all wetlands mapped by the NWI in North Dakota and South Dakota that were subsequently converted to a basin classification system (see Johnson and Higgins 1997). Resulting estimates represent the estimated average number of breeding duck pairs that would be expected to occupy the respective wetlands within the impact site during average wetland conditions in the absence of a wind facility. We computed the *pre‐impact density* by averaging these values from all wetlands within the impact site, which resulted in 1.82 duck pairs per wetland for the impact site. *Bird pairs within impact site* was then equal to 198 pairs (109 wetlands × 1.82 pairs per wetland).

*Percent displacement* was determined from data gathered by Loesch et al. (2013), who reported that wind‐energy production negatively impacted breeding‐pair abundance for Blue‐winged Teal, Northern Shoveler, Gadwall, Mallard, and Northern Pintail. The percent of pairs displaced varied by duck species and wetland class and averaged 18% for seasonal wetlands (Table 1). We focused here on seasonal wetlands for two reasons. First, seasonal wetlands attract the highest densities of breeding waterfowl pairs (Reynolds et al. 2006), and second, seasonal wetlands are the most restored class of previously drained wetlands (Loesch et al. 2012). Using this average percent displacement value, the *bird pairs displaced* in this example was equal to 35.6 pairs (198 pairs in impact site × 0.18 displacement).

**Figure 2:**
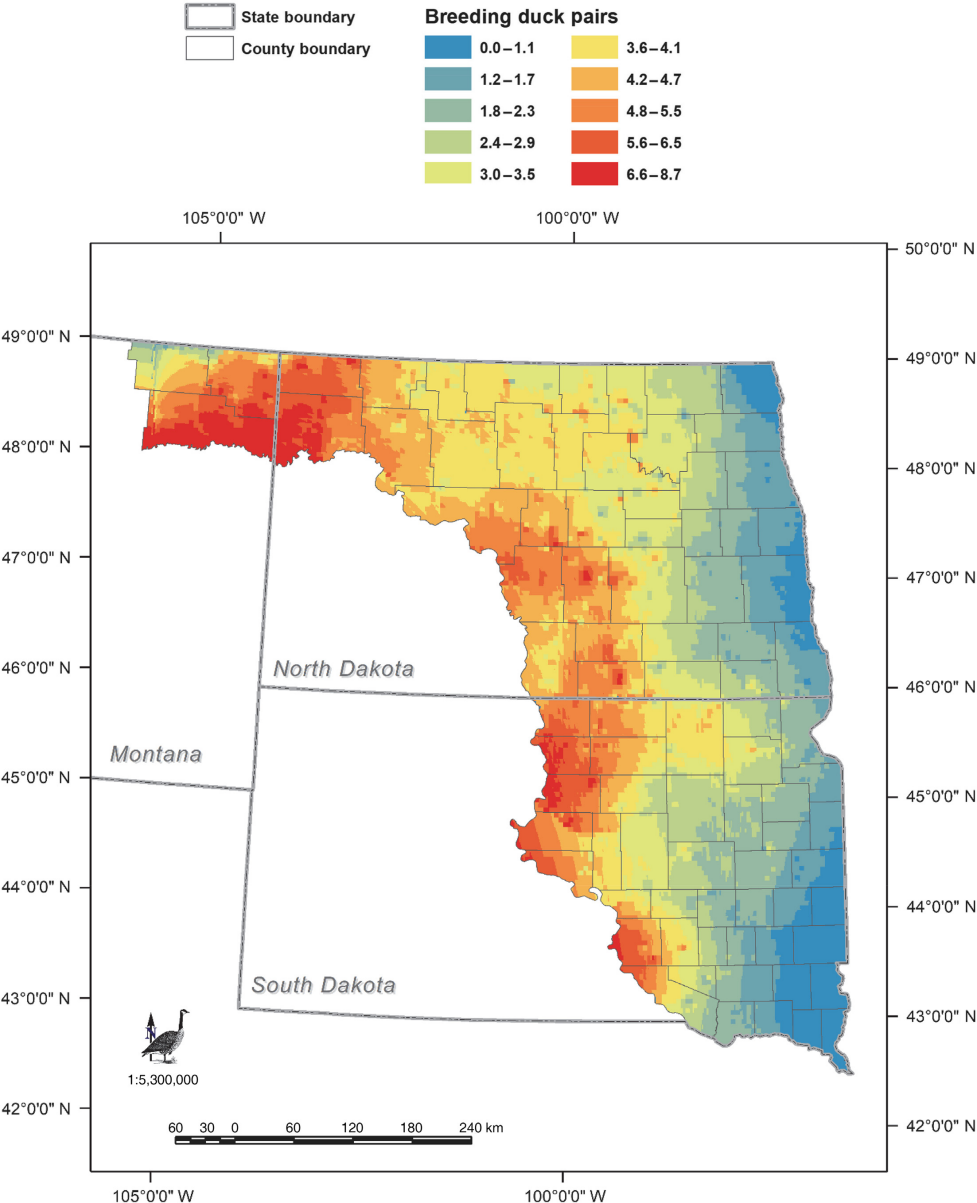
The geographic distribution of the attractiveness of breeding Mallard (*Anas platyrhynchos*), Northern Pintail (*A. acuta*), Blue‐winged Teal (*Spatula discors*), Northern Shoveler (*S. clypeata*), and Gadwall (*Mareca strepera*) pairs to a 0.90‐ha seasonal wetland in the Prairie Pothole Region of North and South Dakota, USA. See Reynolds et al. (2006) for a description of models to estimate breeding duck pairs.

With the objective of estimating the amount of wetland habitat necessary to be restored to provide habitat for the displaced breeding duck pairs, we used the average‐sized seasonal wetland restored by the USFWS Partners for Fish and Wildlife Program (i.e., 0.90 ha) estimated in Loesch et al. (2012) to represent the likely size and class of wetlands to be restored. To account for geographic variation in the attractiveness of a single size and class of wetland to duck pairs (Reynolds et al. 2006), we developed a geospatial data set of 60.8‐ha cells for North Dakota and South Dakota and estimated the breeding pairs for a 0.90‐ha seasonal wetland (range = 0.0–8.7 breeding pairs, Fig. 2) for each cell. The resulting geospatial data allowed us to utilize location‐specific estimates of biological equivalence relative to the location of an impact site. The estimated *offset density* for the hypothetical wind facility is 4.45 pairs per restored seasonal wetland, and the *offset area* equals 8 restored wetlands (35.6 pairs displaced/4.45 pairs per wetland). Using area of the average restored wetland results in a total *wetland area* of 7.2 ha (0.90 ha × 8 wetlands). In summary, eight 0.90‐ha restored seasonal wetlands in the nearby landscape are estimated to provide sufficient offset habitat for the displaced breeding duck pairs (Appendix S1: Table S2).

Within the PPR, spatially explicit models that relate characteristics of wetlands (i.e., size, class, geographical location) with breeding waterfowl abundance have been used for over two decades to evaluate program effectiveness and to develop decision‐support tools (e.g., Thunderstorm Map [Prairie Pothole Joint Venture (PPJV) 2005]) to prioritize the conservation of wetland and grassland habitats that most benefit waterfowl populations based on local and landscape‐level considerations (Reynolds et al. 1996, 2006, PPJV 2005, 2017; Fig. 2). As depicted in Fig. 2, the pair‐abundance models were used to identify locations where restored seasonal wetlands are estimated to harbor higher abundances of breeding duck pairs than seasonal wetlands restored in other locations. Pertinent to the challenge of finding offset sites with equivalent biological value as impact sites, the models can evaluate a pool of potential offset sites in proximity to the impact site to identify sites predicted to be the most similar in biological value to the impact site.

### Example with grassland birds and wind infrastructure

For our grassland bird example, we used the same hypothetical six‐turbine wind facility that we used for the waterfowl example (Fig. 1). We used metrics reported in Shaffer and Buhl (2016), who conducted research that assessed changes in breeding grassland bird density on grazed, mixed‐grass prairies within or adjacent to three wind facilities. We used an *impact distance* of 300 m around each turbine within a wind facility for eight grassland‐obligate bird species as defined by Sauer et al. (2013), as Shaffer and Buhl (2016) found that 87% of the significant displacement effects occurred within 300 m of a turbine. For our hypothetical wind facility, this resulted in an *impact area* of 112 ha.

Using the predicted densities for the eight grassland‐obligate bird species (i.e., Upland Sandpiper [*Bartramia longicauda*], Savannah Sparrow [*Passerculus sandwichensis*], Vesper Sparrow [*Pooecetes gramineus*], Grasshopper Sparrow [*Ammodramus savannarum*], Clay‐colored Sparrow [*Spizella pallida*], Chestnut‐collared Longspur [*Calcarius ornatus*], Western Meadowlark [*Sturnella neglecta*], and Bobolink [*Dolichonyx oryzivorus*]) presented in Shaffer and Buhl (2016), we summed the species densities for the reference sites at each wind facility each year, and then computed an average of all wind facilities and years. We used this average density of 1.9 pairs/ha for the *pre‐impact density* of the hypothetical wind facility, because our hypothetical wind facility was placed in a similar habitat and landscape composition as the study reference sites. *Bird pairs within impact site* was then equal to 213 pairs (112 ha × 1.9 pairs/ha).

We estimated a combined percent displacement value for the eight bird species using predicted bird densities calculated by Shaffer and Buhl (2016) (Appendix S2: Table S1, Step 1). We assumed that any change in density from the pre‐treatment to the post‐treatment years in the reference sites reflected normal annual variation in the bird population, and that we could expect comparable changes in the turbine sites if turbines were not present. Any change above the expected change was ascribed to a turbine effect. To compute percent displacement, we first computed an expected density by distance category for each year post‐treatment based on the percent change in density from pre‐ to post‐treatment for the reference sites (Appendix S2: Table S1, Step 2). We next computed the difference between the predicted density and the expected density for each distance category and year post‐treatment (Appendix S2: Table S1, Step 3). This difference is assumed to be the number of birds displaced by the wind facility within that distance category and year post‐treatment. We then computed the percent displaced by dividing this difference by the expected density for each distance category by time‐period combination and multiplying by 100 (Appendix S2: Table S1, Step 4).

To calculate an average percent displacement, the percent displacement from each of the three wind facilities was averaged for each distance band by time period combination (Appendix S2: Table S1, Step 5). See Appendix S2: Table S2 for an example using Shaffer and Buhl (2016) data from the SD Wind Energy Center. We estimated weighted‐average percent displacement values ranging from 18% in the first year after turbine construction to 53% by the fifth year after turbine construction (Table 2; see Appendix S2: Table S3 for percent displacement values by individual wind facility). The lowest value was 5.69% in the first year post‐construction within 200–300 m from turbines, and the highest value was 59.85% by the fifth year post‐construction within 100 m. These values represent cumulative effects of the wind facility rather than yearly effects. For example, the percent displacement value for 3‐yr post‐treatment is not the displacement from 2 yr to 3 yr post‐treatment; rather, the value is the displacement after the turbines have been in place for 3 yr. Our data cannot be used to extrapolate displacement effects beyond five years post‐construction. For this hypothetical wind facility, we used 53% as the *percent displacement* value, reflecting displacement by the fifth year after turbine construction. We multiplied *bird pairs within impact site* by the *percent displacement* and estimated *bird pairs displaced* to be 113 pairs (213 pairs × 0.53 displacement).

For this example, we will first assume that the offset habitat is a similar habitat type and land use as the impact habitat (i.e., native mixed‐grass prairie that is grazed, located in a similar geographical location with similar topography), and that the *offset density* needed to compensate for displaced pairs is equal to the *pre‐impact density* of 1.9 pairs/ha. The *offset area* is then equal to 59 ha (113 pairs displaced/[1.9 pairs/ha]). Since we assumed that *offset density* is equal to *pre‐impact density*, the amount of habitat needed for the displaced pairs also can be computed as the *impact area* multiplied by *percent displacement*, as stated earlier (i.e., 112 ha × 0.53 displacement = 59 ha; Appendix S1: Table S3).

If we did not assume that the biological value of the offset site was equal to the biological value of the impact site, but rather assumed a situation in which the only available offset habitat was a different habitat, such as a restored grassland, we would need to determine bird density for the restored grassland. For this example, we assumed a hypothetical *offset density* of 1.5 pairs/ha. The amount of habitat needed to support the displaced bird pairs would then be 75 ha of restored grassland (113 pairs/[1.5 pairs/ha]).

These two applications of the model, one case where the biological value (i.e., bird density) of the offset site is equal to the impact site and one case where it is not equal, demonstrates that if a comparably equal offset site cannot be located, more habitat may be needed (e.g., 59 vs. 75 ha in our example) to compensate for bird pairs displaced.

Similar to the intent of the spatially explicit waterfowl models, spatial models that relate landscape, precipitation, topographical, and survey‐specific metrics to relative occurrence probabilities for grassland birds for the prediction of distribution of focal species have been developed for the Northern Great Plains from models utilizing North American Breeding Bird Survey (BBS) data (Niemuth et al. 2017). Pertinent to the challenge of finding offset sites with equivalent biological value as impact sites, the grassland bird models can be used to evaluate a pool of potential offset sites in proximity to the impact site to identify sites predicted to have similar biological value to the impact site. Given that abundance estimates are correlated with estimates of probability of occurrence (Table 3 in Niemuth et al. 2017), the results of the probability of occurrence models in Niemuth et al. (2017) can represent the abundance of grassland birds, and models are available for six of the eight grassland‐obligate species used in our grassland bird and wind infrastructure example (i.e., Upland Sandpiper, Savannah Sparrow, Grasshopper Sparrow, Clay‐colored Sparrow, Western Meadowlark, and Bobolink).

**Figure 3:**
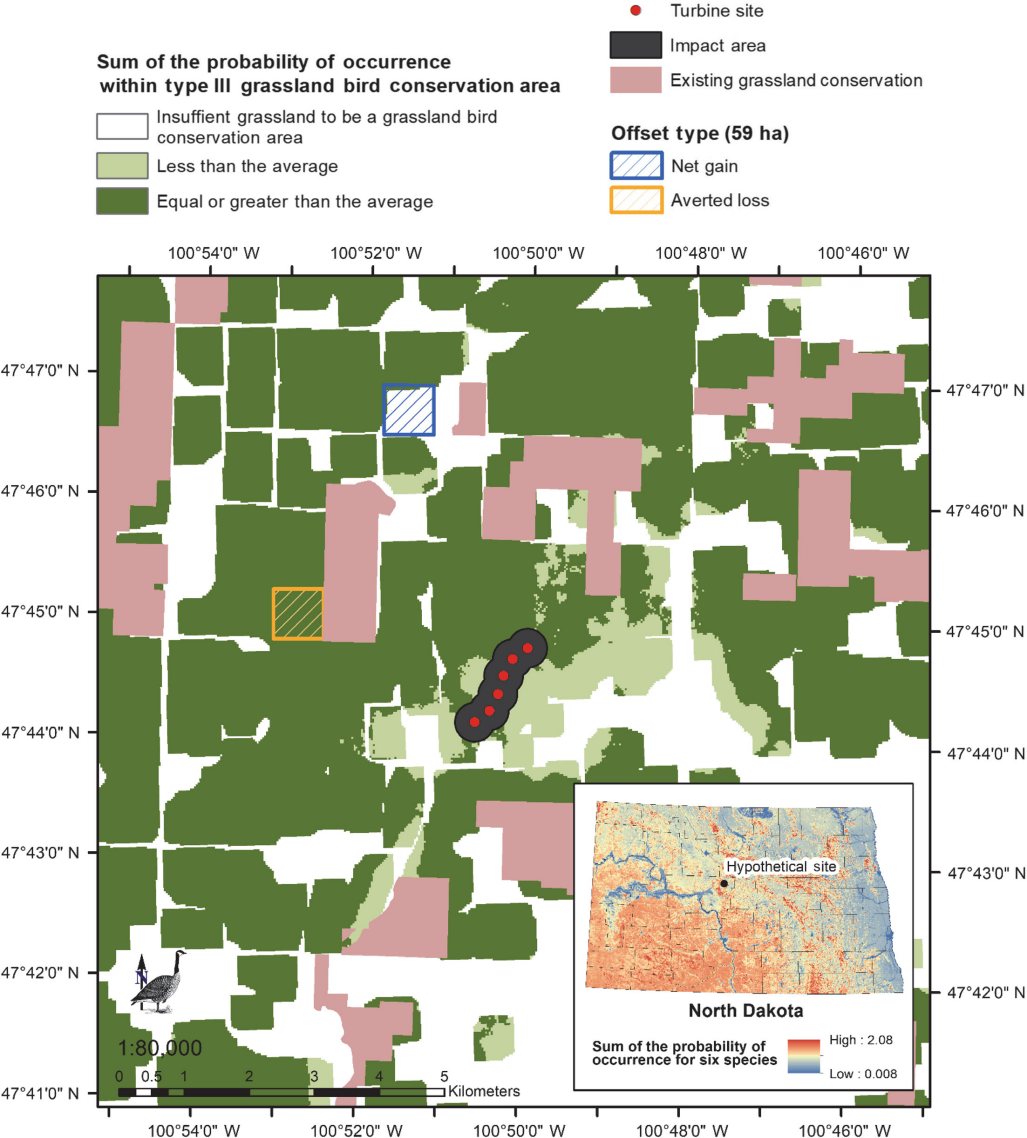
Locations of potential offset sites for grassland bird species displaced by a hypothetical wind facility in the Prairie Pothole Region, USA. Areas shaded with dark green represent locations that meet the landscape composition of a Type III Grassland Bird Conservation Area (Johnson et al. 2010) and where the cumulative probability of occurrence for Upland Sandpiper (*Bartramia longicauda*), Savannah Sparrow (*Passerculus sandwichensis*), Grasshopper Sparrow (*Ammodramus savannarum*), Clay‐colored Sparrow (*Spizella pallida*), Western Meadowlark (*Sturnella neglecta*), and Bobolink (*Dolichonyx oryzivorus*) that is estimated using models from Niemuth et al. (2017) is equal to or exceeds the average probability of occurrence for the six species in the hypothetical wind facility (inset map). Because the average probability of occurrence for the site is used as the threshold for identifying alternative sites for averted loss consideration, portions of the area in the wind facility will display as equal to or higher (dark green), and lower (light green) than the mean. For the grassland bird example in which 59 ha of habitat of comparable biological value is estimated to be necessary to offset behavioral impacts, the protection of 59 ha within the areas shaded dark green will result in an equivalent averted loss offset. Reconstruction of 59 ha of cropland will result in net gain offset if equal biological value is assumed for the reconstructed site.

We used the models from Niemuth et al. (2017) to develop a prototype decision‐support tool for grassland birds using the results of the avian‐impact offset method. We used 30 × 30 m grid results from the Niemuth et al. (2017) models for the six species and summed the occurrence values to identify a cumulative value for each grid cell in North Dakota. We then compared the average of the cumulative cell values within the hypothetical wind facility and its associated impact area to the cumulative cell values in the rest of the state. Locations where the cumulative cell values are equal or exceed the average value of the impact site are considered potential offset locations (Fig. 3). Finally, we limited the potential offset locations to areas included in Type III Grassland Bird Conservation Areas as defined in Johnson et al. (2010), which served to eliminate small, fragmented patches of grassland from consideration as offset sites. In the context of our grassland bird example in which the amount of habitat needed to offset the displaced grassland bird pairs was 59 ha, any 59‐ha location shaded dark green in Fig. 3 would be considered an equivalent offset location for averted‐loss acquisition. Thus, it is feasible to translate the results of the avian‐impact offset method to the development of decision‐support tools that inform landscape‐level conservation delivery of offsetting measures.

### Example with grassland birds and oil‐extraction infrastructure

For an example in applying the avian‐impact offset method to oil and gas infrastructure, we use the results of Thompson et al. (2015), who measured avoidance of unconventional oil wells for 10 species of grassland birds in mixed‐grass prairies in northwestern North Dakota. We used information provided in Figure 4D of Thompson et al. (2015) as an example of a hypothetical oil‐extraction development. In that example, the *impact distance* was 350 m for oil wells and 150 m for roads. The figure depicts a 2,048‐ha study area of grassland in which are placed 14 wells and associated roads buffered by their respective impact distances. The authors found that 46% of the study area was impacted, so the *impact area* is 942 ha (2,048 × 0.46). *Pre‐impact density* was reported as 3.182 pairs/ha (Appendix A in Thompson et al. 2015). The authors cite a *percent displacement* of 33%. To simplify the calculations, we assumed that the offset habitat is biologically equivalent to the impact habitat so that the *pre‐impact density* and the *offset density* are the same value. As a result, we estimated that the amount of mixed‐grass prairie habitat necessary to replace the grassland bird pairs displaced by oil wells and roads in this particular scenario was 311 ha (942 ha × 0.33 displacement) (Appendix S1: Table S4).

## Discussion

Our avian‐impact offset method provides an approach to quantifying the impact of behavioral avoidance to energy infrastructure and other forms of anthropogenic development in such a way that the impact can inform offset decisions with meaningful on‐the‐ground conservation actions. The choice of birds as the biological indicator taxon has advantages over other taxa because birds are visually or aurally conspicuous, the number of species an observer must learn to identify in any given geographical location is manageable, survey methodology has a long‐established history of scrutiny and technological advancement (Ralph et al. 1993, Sutherland et al. 2004), and avian density is an easily measured metric. Floristic quality assessments measure a natural area's ecological integrity (Freyman et al. 2016), but plant species composition can vary widely over small geographical distances, and thus plant identification can be daunting and the likelihood of finding offset sites of equivalent biological value, based on plant composition, is challenging.

Because avian density may be a misleading indicator of habitat quality (Van Horne 1983), the addition of fitness metrics would better support the assumption that an offset site provides equivalent biological value for breeding birds. If fitness data are available and relate to a measure of area (i.e., hectares), the pre‐ and post‐impact density could be substituted with that metric. A combination of density and fitness metrics would begin to address the concern of simplifying the multi‐dimensional values of biodiversity and complex ecological processes and interactions in native ecosystems to a single metric, and the problem that values that are not defined are thus not reflected in offsets, which has been documented (Moreno‐Mateos et al. 2015). However, the more complex the values of biological equivalency, the more difficult it is to convert to a common metric that can be exchanged between geographical locations and to measure success or failure, and so these concerns reduce the likelihood of implementation of offsetting in general (Goncalves et al. 2015), as does the cost of acquiring data from long‐term and expensive field‐based studies.

We believe our method balances both complexity and utility and can be used where mitigation is focused toward on‐the‐ground conservation. To that end, in our method, impact is ultimately expressed as habitat area (i.e., hectares of habitat), which is a metric easily understood by the public, private landowners, legislators, regulators, and taxation agencies. Habitat area is the traditional land‐unit metric by which conservation lands are purchased (USFWS 2016) and for which conservation banking credits are calculated under methods such as the Landscape Equivalency Analysis (Bruggeman et al. 2005).

The avian‐impact offset method requires knowledge of several biological parameters relating to the influence of anthropogenic disturbance on birds, including the distance that the disturbance exerts an influence on a bird, the total area thus influenced, the expected breeding‐pair density on the impacted site before the disturbance occurs, the displacement rate after disturbance, and optimally an expected breeding‐pair density on the potential offset site. Therefore, the avian‐impact offset method has several metrics that must be measured or assumed for the equivalent biological value of the impact to be estimated. The distance that infrastructure exerts an influence on bird behavior must be obtained. Several authors have reported discrete displacement distances based on their design and analysis (Winkelman 1992, Pearce‐Higgins et al. 2009, Garvin et al. 2011, Sansom et al. 2016, Shaffer and Buhl 2016, Fernandez‐Bellon et al. 2018). Yet other authors express displacement distance as a continuous variable without author interpretation of a specific distance (Kalyn Bogard and Davis 2014).

The density estimate that is required of the impact and offset sites is a composite of the focal‐species densities, and thus users of our method must consider the composition of bird species of the impact and offset sites. Ideally, the species composition on the offset site would be comparable to the impact site, but this may not be possible in all cases. A similar avian density on the offset site as on the impact site may be achievable, but it may be based on a species composition and individual‐species densities that may differ somewhat from that occurring on the impact site. For example, the offset site could contain all but one of the bird species on the impact site, or the offset site could harbor slightly lower densities for some species but higher densities for other species. It is critical that the possibility for these potential differences is articulated and discussed by the relevant parties. Our method does not account for variations in individual‐species density; it only focuses on the composite density for the focal species.

The percent displacement metric accounts for the fact that not all individuals of a species abandon the area near anthropogenic infrastructure. A limited number of authors have published values for percent displacement (Table 1, Table 2; Pearce‐Higgins et al. 2009, Garvin et al. 2011, Pearce‐Higgins et al. 2012, Thompson et al. 2015, Sansom et al. 2016) that may be pertinent to users in some situations, for example, where the bird species and habitat are the same, and thus, may be used to inform estimates of percent displacement. If, for example, wind‐energy development is planned for a location where the range for species with published avoidance information exists but the habitat characteristics differ (e.g., mixed grass prairie vs. sagebrush), an assumption could be made that the percent displacement for the respective species would be similar, and estimates for habitat with equivalent biological value can be estimated. It is critical that this assumption is articulated and agreed upon by the relevant parties. However, if the assumptions are deemed inappropriate or unacceptable, a more appropriate value for percent displacement must be calculated from field‐based research or alternative data sets.

There are several considerations in choosing an offset site, depending on whether the focal species of concern are waterfowl or grassland birds, the type of offset being pursued, and the location of the offset site relative to landscape factors as defined earlier. In our grassland bird examples, the offset site was existing grassland habitat and thus an “averted‐loss” biodiversity offset, in which there is no net biodiversity gain, but rather a compensated net loss with a guarantee of no future development (i.e., protection; Curran et al. 2014). We used an averted‐loss scenario because, in the PPR, most remaining native temperate grasslands and wetlands are in private ownership (Doherty et al. 2013). In the PPR, averted loss has been administered by conservation professionals and often results in federal easements (see Loesch et al. 2013, Claassen et al. 2017) to protect native habitats that may otherwise be converted to agricultural or other purposes. The advantage of protecting native habitats is that it protects an entire ecosystem, not just the metric that was measured. Another advantage with averted loss is that one could eliminate the need to use offset multipliers. Multipliers are the ratios between damaged and compensated amounts of biodiversity and are often employed to inform decisions relative to the amount of offset needed (Laitila et al. 2014). However, the exact values of multipliers are difficult to compute and even the absolute minimum values may be quite large, despite the relatively low multipliers actually found in practice (Laitila et al. 2014). Multipliers are subjective and are commonly used to place a higher societally derived value on one habitat type over another; for example, in state wind‐siting guidelines, unbroken (native) grasslands are deemed of higher intrinsic value than restored native grasslands, and thus must be mitigated at a higher multiplier level (NWWWG 2016).

Practitioners of our method may choose to pursue other types of offsets, such as ones that achieve net biodiversity neutrality or gain. To achieve net biodiversity neutrality or gain, grassland reconstruction is necessary, and the biodiversity or ecological functions lost in the developed area must be replicated with equal or greater measure in the newly created habitat. A common criticism of this offset approach is that it exchanges certain and almost immediate losses for uncertain future gains, which for some habitats, are in the timeframe of decades or even centuries (Laitila et al. 2014). With habitat reconstructions, there is higher uncertainty in what species will eventually inhabit the offset site post‐construction, and in what density, factors that are less uncertain in an averted‐loss scenario in which the offset habitat currently exists, and in which species composition, both avian and floral, have remained relatively consistent over time. Grassland reconstruction necessitates consideration of the several factors of uncertainty, namely time lags, potential for restoration failure and failure to persist, and measurability (Maron et al. 2012, Curran et al. 2014, Laitila et al. 2014). Uncertainty factors are the foundation for the development and application of multipliers. Because multipliers are often assigned based on policy considerations and not on scientific merit, they are beyond the scope of this paper. Despite the challenges of reconstructing habitats, planted grasslands have a well‐established history of providing avian breeding habitat, as witnessed by the acknowledged benefits to birds of the USDA Conservation Reserve Program (Herkert 2009, Allen and Vandever 2012), and should not be dismissed out of hand. The decision of whether to apply an averted‐loss scenario or a net‐neutral scenario will be up to the practitioners of our method and their ultimate conservation goals.

For waterfowl, we assume that restoring the hydrologic function to a previously drained wetland results in equal biological benefits for breeding waterfowl pairs to wetlands that have no drainage history. In our waterfowl example, our method estimates the number and characteristics of wetlands (i.e., size, wetland class) that, if restored, are necessary to provide biodiversity offsets. The offset wetlands are assumed to represent net neutral biodiversity offset, because areas of hydric soils with a history of recent past conversion from wetland to agricultural use are typically restored (USFWS 2017), and wetland function returns with restored hydrology (Wienhold and van der Valk 1989, Galatowitsch and van der Valk 1996). Consequently, from an offset perspective, we assume that the wetlands with restored hydrologic function provide biological equivalency for breeding waterfowl pairs relative to wetlands that have no drainage history. Similar to the situation with reconstructed grasslands, the assignment of multipliers to compensate for uncertainty factors is beyond the scope of this paper.

The question of where to establish offset sites that maximize conservation value on the landscape can be addressed with the application of decision‐support tools for waterfowl and grassland birds. These tools identify the locations of grasslands or wetlands predicted to contain waterfowl and grassland birds in equivalent numbers to offset sites. Furthermore, the models can predict locations where existing biological value will be so high that developers of energy‐production facilities and other types of disturbance may decide to adhere to the first principle of the mitigation hierarchy and avoid that particular area.

The avian‐impact offset method described herein provides conservation professionals and developers of energy and transportation infrastructure, as well as developers of other forms of anthropogenic infrastructure, a science‐based tool that calculates the biological value (in this method, avian density) lost by development. The method's output (e.g., number of wetlands or hectares of grassland with specified characteristics) converts biological value to the traditional unit of measure in which land is purchased or sold in the conservation community. The areal unit of measure also lends itself readily to mapping applications in which conservation delivery of offsetting measures can be viewed at local, regional, or landscape scales. The avian‐impact offset method evaluates behavioral impacts on one specific parameter affecting bird populations, that is, density. Future efforts should focus on quantifying other types of impacts, such as nest survival, adult survival, recruitment of young, and bird migration. The decision‐support tools identify locations for placement of offset sites that are most likely to meet or exceed the biological values on the impact sites and identify locations that would require a high biodiversity offset cost if developed, relative to other potential locations. Our methodology represents a starting point for offset discussions between developers and those responsible for conserving and managing habitat for wildlife.

## Data Availability

All data used in the ANOVA models to predict the grassland bird densities that were used to compute the percent displacement values for grassland birds are publicly available through USGS ScienceBase at https://doi.org/10.5066/F7T43SDG.
