# Supplementary material for: Estimating offsets for avian displacement effects of anthropogenic impacts
Source: Ecol Appl. 2019 Aug 30;29(8):e01983. doi: 10.1002/eap.1983 (PMC6916566; doi:10.1002/eap.1983)
Supplement: Supplementary file 1 [file EAP-29-na-s001.pdf]

Supporting Information. Shaffer, J. A., C. R. Loesch, and D. A. Buhl. 2019. Estimating offsets for avian displacement effects of anthropogenic impacts. *Ecological Applications.*

Appendix S1

Table S1. Calculation sheet developed to use the avian-impact offset method to estimate offsets for displaced breeding bird pairs.

					Row 1
					2
Instructions:					3
1. Fill in Metric (i.e., numeric value for the parameter), Units, and Source column for rows 10-14.					4
2. If density is measured as number pairs per hectare, Impact Area should be measured in hectares. If density is measured as number pairs per wetland, Impact Area should be measured as number of wetlands.					5
3. Enter Percent Displacement as a number between 1 and 100.					6
4. The units for the Offset Area will be the area units from the Offset Density, e.g., if Offset Density is measured as pairs/ha then Offset Area will be ha and if Offset Density is measured as pairs/wetland then Offset Area is number of wetlands.					7
					8
Parameter	Metric	Units	Source	Formula	9
Impact Distance					10
Impact Area					11
Pre-Impact Density					12
Percent Displacement		percent			13
Offset Density					14
Number Pairs in Impact Site		***pairs***		***B15 = B11*B12***	15
Number Pairs Displaced		***pairs***		***B16 = B15*(B13/100)***	16
Offset Area				***B17 = B16/B14***	17
Column A	B	C	D	E	

Appendix S1. Table S2. Example 1. Calculation sheet developed to use the avian-impact offset method to estimate offsets for displaced breeding waterfowl pairs using hypothetical 6-turbine wind facility example.

					Row
Appendix S1. Table S2. Example 1. Calculation sheet developed to use the avian-impact offset method to estimate offsets for displaced breeding waterfowl pairs using hypothetical 6-turbine wind facility example.					1
					2
Instructions:					3
1. Fill in Metric (i.e., numeric value for the parameter), Units, and Source column for rows 10-14.					4
2. If density is measured as number pairs per hectare, Impact Area should be measured in hectares. If density is measured as number pairs per wetland, Impact Area should be measured as number of wetlands.					5
3. Enter Percent Displacement as a number between 1 and 100.					6
4. The units for the Offset Area will be the area units from the Offset Density, e.g., if Offset Density is measured as pairs/ha then Offset Area will be ha and if Offset Density is measured as pairs/wetland then Offset Area is number of wetlands.					7
					8
Parameter	Metric	Units	Source	Formula	9
Impact Distance	0.8	km	Loesch et al. (2013)		10
Impact Area	109	wetlands	Derived from GIS		11
Pre-Impact Density	1.82	pairs/wetland	Reynolds et al. (2006)		12
Percent Displacement	18	percent	This paper		13
Offset Density	4.5	pairs/wetland	Loesch et al. (2012)		14
Number Pairs in Impact Site	***198***	***pairs***		***B15 = B11*B12***	15
Number Pairs Displaced	***36***	***pairs***		***B16 = B15*(B13/100)***	16
Offset Area	***8***	***wetlands***		***B17 = B16/B14***	17
Column A	B	C	D	E	

Loesch, C. R., R. E. Reynolds, and L. T. Hansen. 2012. An assessment of re-directing breeding waterfowl conservation relative to predictions of climate change. Journal of Fish and Wildlife Management 3:1-22.

Loesch, C. R., J. A. Walker, R. E. Reynolds, J. S. Gleason, N. D. Niemuth, S. E. Stephens, and M. A. Erickson. 2013. Effect of wind energy development on breeding duck densities in the Prairie Pothole Region. Journal of Wildlife Management

Reynolds, R. E., T. L. Shaffer, C. R. Loesch, and R. R. Cox, Jr. 2006. The farm bill and duck production in the Prairie Pothole Region: increasing the benefits. Wildlife Society Bulletin 34:963-974.

Appendix S1. Table S3. Example 2. Calculation sheet developed to use the avian-impact offset method to estimate offsets for displaced breeding grassland bird pairs using hypothetical 6-turbine wind facility example. Row 1

					2
Instructions:					3
1. Fill in Metric (i.e., numeric value for the parameter), Units, and Source column for rows 10-14.					4
2. If density is measured as number pairs per hectare, Impact Area should be measured in hectares. If density is measured as number pairs per wetland, Impact Area should be measured as number of wetlands.					5
3. Enter Percent Displacement as a number between 1 and 100.					6
4. The units for the Offset Area will be the area units from the Offset Density, e.g., if Offset Density is measured as pairs/ha then Offset Area will be ha and if Offset Density is measured as pairs/wetland then Offset Area is number of wetlands.					7
					8
Parameter	Metric	Units	Source	Formula	9
Impact Distance	300	m	Shaffer and Buhl (2016)		10
Impact Area	112	ha	Derived from GIS		11
Pre-Impact Density	1.9	pairs/ha	Shaffer and Buhl (2016)		12
Percent Displacement	53	percent	This paper		13
Offset Density	1.9	pairs/ha	Equal Value Habitat		14
Number Pairs in Impact Site	***213***	***pairs***		***B15 = B11*B12***	15
Number Pairs Displaced	***113***	***pairs***		***B16 = B15*(B13/100)***	16
Offset Area	***59***	***ha***		***B17 = B16/B14***	17
Column A	B	C	D	E	

Shaffer, J. A., and D. A. Buhl. 2016. Effects of wind-energy facilities on grassland bird distributions. Conservation Biology 30:59-71.

Appendix S1. Table S4. Example 3. Calculation sheet developed to use the avian-impact offset method to estimate offsets for displaced breeding grassland bird pairs using an oil-extraction infrastructure example.					Row 1
					2
Instructions:					3
1. Fill in Metric (i.e., numeric value for the parameter), Units, and Source column for rows 10-14.					4
2. If density is measured as number pairs per hectare, Impact Area should be measured in hectares. If density is measured as number pairs per wetland, Impact Area should be measured as number of wetlands.					5
3. Enter Percent Displacement as a number between 1 and 100.					6
4. The units for the Offset Area will be the area units from the Offset Density, e.g., if Offset Density is measured as pairs/ha then Offset Area will be ha and if Offset Density is measured as pairs/wetland then Offset Area is number of wetlands.					7
					8
Parameter	Metric	Units	Source	Formula	9
Impact Distance	350/150	m; wells/roads	Thompson et al. (2015)		10
Impact Area	942	ha	Thompson et al. (2015)		11
Pre-Impact Density	3.2	pairs/ha	Thompson et al. (2015)		12
Percent Displacement	33	percent	Thompson et al. (2015)		13
Offset Density	3.2	pairs/ha	Equal Value Habitat		14
Number Pairs in Impact Site	***3014***	***pairs***		***B15 = B11*B12***	15
Number Pairs Displaced	***995***	***pairs***		***B16 = B15*(B13/100)***	16
Offset Area	***311***	***ha***		***B17 = B16/B14***	17
Column A	B	C	D	E	

Thompson, S. J., D. H. Johnson, N. D. Niemuth, and C. A. Ribic. 2015. Avoidance of unconventional oil wells and roads exacerbates habitat loss for grassland birds in the North American Great Plains. Biological Conservation 192:82-90.
