# Supplementary material for: Estimating offsets for avian displacement effects of anthropogenic impacts
Source: Ecol Appl. 2019 Aug 30;29(8):e01983. doi: 10.1002/eap.1983 (PMC6916566; doi:10.1002/eap.1983)
Supplement: Supplementary file 2 [file EAP-29-na-s002.pdf]

Supporting Information. Shaffer, J. A., C. R. Loesch, and D. A. Buhl. 2019. Estimating offsets for avian displacement effects of anthropogenic impacts. *Ecological Applications*.

Appendix S2

Appendix S2: Table S1. Steps to compute values of percent displacement from wind turbines for grassland breeding pairs and the variable definitions.

**Steps to compute values of percent displacement for grassland breeding pairs**	**Equation variable definitions**
**Step 1:**<br>$TotPDens_{hij} = \sum_k PDens_{hijk}$<br>For all *h*, *i*, *j*, and *k*	*h* = wind facility number = 1, 2, or 3 (arbitrary number assigned to a wind facility)<br><br>*i* = time period indicator = 0 (pre-trt), 1 (1-yr post), 2 (2-yr post), 3 (3-yr post), or 4 (5-yr post)<br><br>*j* = treatment or distance category indicator = 0 (reference), 1 (0-100 m from turbines), 2 (100-200 m from turbines), and 3 (200-300 m from turbines)
**Step 2:**<br>$Exp_{hij} = \frac{TotPDens_{hi0}}{TotPDens_{h00}} \times TotPDens_{h0j}$<br>For all *h*, *i* > 0, *j* > 0	*k* = species number = 1, 2, 3, 4, 5, 6, 7, or 8 (arbitrary number assigned to species)<br><br>$PDens_{hijk}$ = predicted density (per 100 ha) for species *k* for time period *i* and distance category *j* at wind facility *h* (predicted using the ANOVA models from Shaffer and Buhl 2016[a])
**Step 3:**<br>$Diff_{hij} = TotPDens_{hij} - Exp_{hij}$<br>For all *h*, *i* > 0, *j* > 0	$TotPDens_{hij}$ = sum total predicted density of 8 grassland species for time period *i* and distance category *j* at wind facility *h*
**Step 4:**<br>$PercDisp_{hij} = \frac{Diff_{hij}}{Exp_{hij}} \times 100$<br>For all *h*, *i* > 0, *j* > 0	Exp_{hij} = expected density (per 100 ha) for 8 grassland species for time period *i* and distance category *j* at wind facility *h*<br><br>$Diff_{hij}$ = difference between total predicted density and expected density (i.e., the number of birds/100 ha displaced) for time period *i* (*i*>0) and distance category *j* (*j*>0) at wind facility *h*
**Step 5:**<br>$AvePercDisp_{ij} = \frac{\sum_h PercDisp_{hij}}{3}$<br>For all *h*, *i* > 0, *j* > 0	$PercDisp_{hij}$ = percent of birds/100 ha displaced for time period *i* (*i*>0) and distance category *j* (*j*>0) at wind facility *h*<br><br>$AvePercDisp_{ij}$ = average percent displaced for time period *i* (*i*>0) and distance category *j* (*j*>0)

[a] Shaffer, J. A., and D. A. Buhl. 2016. Effects of wind-energy facilities on grassland bird distributions. Conservation Biology 30:59-71. DOI: 10.1111/cobi.12569.

Appendix S2: Table S2. Example calculations for estimating percent displacement from wind turbines for each time period by distance combination by wind facility for eight grassland bird species[a], using the predicted densities per 100 ha from the ANOVA models in Shaffer and Buhl (2016)[b] for the South Dakota Wind Energy Center during the first year after turbine construction.

**Step 1: Sum predicted density/100 m across 8 species.**				
**Time Period**	**0-100 m**	**10-200 m**	**200-300 m**	**Reference**
Pre-trt	232.64	193.34	231.99	213.84
1-year post-trt	83.97	86.56	113.40	138.68
**Step 2: Compute expected density for turbine sites by distance category.**				
1-year post-trt	150.87	125.38	150.45	
**Step 3: Compute difference between predicted and expected density.**				
1-year post-trt	-66.90	-38.82	-37.05	
**Step 4: Compute percent displaced.**				
1-year post-trt	-44.34%	-30.96%	-24.63%	

Step 2 (0-100 m):

$$\frac{138.68}{213.84} \times 232.64 = 150.87$$

Step 3 (0-100 m):

$$83.97 - 150.87 = -66.90$$

Step 4 (0-100 m):

$$\frac{-66.90}{150.87} \times 100 = -44.34\%$$

[a] The eight grassland bird species are Upland Sandpiper (*Bartramia longicauda*), Savannah Sparrow (*Passerculus sandwichensis*), Vesper Sparrow (*Pooecetes gramineus*), Grasshopper Sparrow (*Ammodramus savannarum*), Clay-colored Sparrow (*Spizella pallida*), Chestnut-collared Longspur (*Calcarius ornatus*), Western Meadowlark (*Sturnella neglecta*), and Bobolink (*Dolichonyx oryzivorus*).

[b] Shaffer, J. A., and D. A. Buhl. 2016. Effects of wind-energy facilities on grassland bird distributions. Conservation Biology 30:59-71. DOI: 10.1111/cobi.12569.

Appendix S2: Table S3. Average percent displacement (standard error) for bird species by distance category, year post-treatment, and wind facility for three wind facilities placed in grazed mixed-grass prairies in North Dakota, USA (Acciona Tatanka Wind Farm and NextEra Energy Oliver Wind Energy Center) and South Dakota, USA (NextEra Energy SD Wind Energy Center), 2003-2012[a]. Average column is a weighted average of the values of percent displacement for the 3 distance bands in that row; areas of the distance bands were used as the weights.

**Years post-treatment**	**<100 m**	**100-200 m**	**200-300 m**	**Average**
**Acciona Tatanka Wind Farm[b]**				
2	40.43 (17.84)	47.44 (16.61)	32.66 (15.77)	38.45 (10.55)
3	43.85 (15.82)	38.60 (14.27)	38.57 (14.01)	39.17 (9.29)
5[c]	63.66 (15.70)	50.23 (13.77)	45.40 (13.33)	49.04 (8.89)
**NextEra Energy Oliver Wind Energy Center[d]**				
1	58.74 (23.88)	23.15 (22.22)	-- -- [e]	6.89 (13.95)
3	72.55 (17.79)	60.76 (17.08)	43.94 (15.27)	52.73 (10.41)
5[c]	37.24 (19.98)	58.69 (21.22)	49.62 (19.56)	51.27 (13.16)
**NextEra Energy South Dakota Wind Energy Center[f]**				
1	44.34 (19.78)	30.96 (20.80)	24.63 (18.95)	28.93 (12.80)
2	47.29 (22.71)	36.79 (23.79)	31.98 (21.87)	35.28 (14.73)
3	55.49 (17.08)	24.77 (16.91)	35.93 (16.08)	34.38 (10.73)
5[c]	78.67 (16.13)	65.17 (16.36)	50.33 (14.37)	58.42 (9.83)

[a] Average percent displacement values are based on the predicted densities per 100 ha from the ANOVA models in Shaffer and Buhl (2016). Shaffer, J. A., and D. A. Buhl. 2016. Effects of wind-energy facilities on grassland bird distributions. Conservation Biology 30:59-71. DOI: 10.1111/cobi.12569.

[b] No data were gathered for 1- or 4-years post-treatment. Data are for seven grassland birds species: Upland Sandpiper (UPSA) (*Bartramia longicauda*); Savannah Sparrow (SAVS) (*Passerculus sandwichensis*); Vesper Sparrow (VESP) (*Pooecetes gramineus*); Grasshopper Sparrow (GRSP) (*Ammodramus savannarum*); Clay-colored Sparrow (CCSP) (*Spizella pallida*);

Western Meadowlark (WEME) (*Sturnella neglecta*); and Bobolink (BOBO) (*Dolichonyx oryzivorus*).

[c] Extrapolation of data beyond 5 years would be inappropriate.

[d] No data were gathered for 2- or 4-years post-treatment. Data are for the same species as listed above.

[e] A positive value of 13.24 (20.74), indicating attraction.

[f] No data were gathered for 4-years post-treatment. Data are for UPSA, GRSP, WEME, BOBO, and Chestnut-collared Longspur (*Calcarius ornatus*).
